# Non-cryopreserved Kx negative packed red cell concentrates to support hematopoietic stem cell transplantation in McLeod contiguous gene deletion syndrome

**DOI:** 10.70962/jhi.20250067

**Published:** 2025-08-11

**Authors:** Julian Thalhammer, Charlotte Engström, Brigitte Strahm, Carsten Speckmann, Ayami Yoshimi, Andreas Scharberg, Elke Weinig, Marie-Christin Pauly, Markus Umhau, Stefan Meyer, Stephan Ehl, Beat M. Frey, Richard Schäfer

**Affiliations:** 1 Center for Pediatrics and Adolescent Medicine, Medical Center - University of Freiburg, Faculty of Medicine, University of Freiburg, Freiburg, Germany; 2 Institute for Immunodeficiency, Center for Chronic Immunodeficiency (CCI) Medical Center - University of Freiburg, Freiburg, Germany; 3 Faculty of Medicine, University of Freiburg, Freiburg, Germany; 4 Blood Transfusion Service Zurich, Swiss Red Cross, Schlieren, Switzerland; 5 German Red Cross Blood Donor Service Baden-Württemberg-Hessen gGmbH Baden-Baden, Baden-Baden, Germany; 6 Institute for Transfusion Medicine and Gene Therapy, Medical Center - University of Freiburg, Freiburg, Germany

## Abstract

Transfusion management of Kx− individuals with McLeod phenotype (MLP) is highly challenging, particularly as cryopreservation affects red blood cell (RBC) concentrate quality. We developed a concept to provide non-cryopreserved Kx− RBCs over the complete period of hematopoietic stem cell transplantation (HSCT) for treatment of X-linked chronic granulomatous disease (X-CGD) with MLP. An infant with a large deletion affecting 12 protein-coding genes, including *DMD*, *PRRG1*, *LANCL3*, *XK*, *CYBB*, and *DYNLT3*, leading to CGD, Duchenne muscular dystrophy, and MLP, was scheduled for HSCT with the need of Kx− blood supply. No Kx− and RhD compatible donors were identified by rare donor programs, and autologous blood collection was not possible. In an interdisciplinary multicenter effort pre- and post-HSCT blood management, including procurement of non-cryopreserved allogeneic Kx− RBCs from an individual with MLP, was orchestrated, balancing donations, storage, pediatric RBC preparation, and irradiation with the clinical schedule. Our concept ensured compatible blood supply from 100 days prior HSCT to the peritransplant phase. The patient received 5 non-cryopreserved Kx− pediatric RBCs and was discharged with complete chimerism at day +68. The screen was repeatedly negative for antibodies to high frequency RBC antigens. After 2.8 years, the patient remained independent of transfusions and was without signs of graft-versus-host disease. Close coordination between institutions and disciplines and process optimization allow readily available provision of non-cryopreserved Kx− RBCs to support HSCT to a patient with unique contiguous gene deletion syndrome of X chromosome.

## Teaser

A large, previously unknown deletion of 12 protein-coding genes in X-linked CGD with MLP was identified. Allogeneic Kx− RBCs were collected from another individual with MLP, and non-cryopreserved RBCs were readily available over several months prior to HSCT to the peritransplant phase.

## Introduction

Chronic granulomatous disease (CGD) is an inborn error of immunity characterized by malfunction of neutrophils and macrophages due to reduced or absent activity of the NADPH oxidase. Patients suffer from recurrent bacterial and fungal infections, granuloma formation, and severe inflammatory complications. Genes relevant for NADPH oxidase function can cause CGD. The most common form is X-linked with mutations in *CYBB* (g91^phox^). Larger deletions of the X chromosome affect nearby genes, referring to as contiguous gene deletion syndrome. Depending on the specific deletion, McLeod syndrome (MLS), Duchenne muscular dystrophy, infertility, and retinitis pigmentosa can occur. MLS is caused by mutations in *XK*, leading to acanthocytosis and reduced/absent expression of Kell blood group antigens due to truncated XK protein with lack of Kx antigen (McLeod phenotype [MLP]) (1). Red blood cells of healthy individuals universally carry the XK protein (Kx+), and Kx− individuals may form anti-RBC alloantibodies (anti-Kx and anti-Km) upon transfusion of Kx+-packed RBC units, which may lead to hemolytic transfusion reactions. Therefore, transfusion management of Kx− individuals is challenging, particularly in the context of hematopoietic stem cell transplantation (HSCT) and considering that cryopreservation affects RBC quality.

Cryopreservation of Kx− RBCs comes with several drawbacks. Frozen RBCs can break during transport and thawing. The latter involves deglycerolizing steps, leading to substantial hemolysis rates of up to 50% and limited RBC shelf life. Also, cryopreservation can negatively impact deformability and membrane rigidity of autologous RBCs in a CGD and MLP setting (2). Therefore, we elaborated an individual RBC manufacture and transfusion concept to supply fresh Kx− RBCs without need for cryopreservation to provide optimal RBC quality for an infant patient during the critical peritransplant phase.

## Case presentation

The patient was the first child of a non-consanguineous family with no relevant medical history. After uneventful pregnancy, birth at term was complicated by neonatal asphyxia (Apgar scores 2 after 5 and 10 min, umbilical artery pH = 7.05). The patient received resuscitation, therapeutic hypothermia, and was on invasive ventilation for 5 days. Central line infection with *Staphylococcus aureus* required intravenous antibiotic therapy. At 2.5 mo of age, severe pneumonia with pleural effusion muscular hypotonia and transaminitis triggered genetic and immunologic workup. At 4 mo of age, the diagnosis of CGD was established, and *Burkholderia multivorans* pneumonia was successfully treated.

Severe anemia (Hb: 6.9 g/dl) was an additional complicating factor. Acanthocytosis was found, MLP was confirmed by flow cytometry, and serology showed weak and absent Kell antigens [K−k+^w^, Kp(a−b−), Js(b−), and Kx−]; Js^a^ was not tested. Further serologic typing showed red blood cell phenotype B, RhD−,C−, c+, E−, e+, C^w^− (rr), Jk(a+b−), Fy(a+b+), S−, s+, M+, N+. The Kell blood group genotype was KEL:−1, 2, −3, 4, −6, and 7 (1). The negative Kp^b^ and Js^b^, and the weak k expression, contrasted to the KEL genotype, whereby the complete negative results likely resulted from weak affinity of the anti-Kp^b^ and anti-Js^b^ test sera. The patient had not received RBC transfusions previously, and screening for irregular red blood cell antibodies was negative.

**Figure 1.**
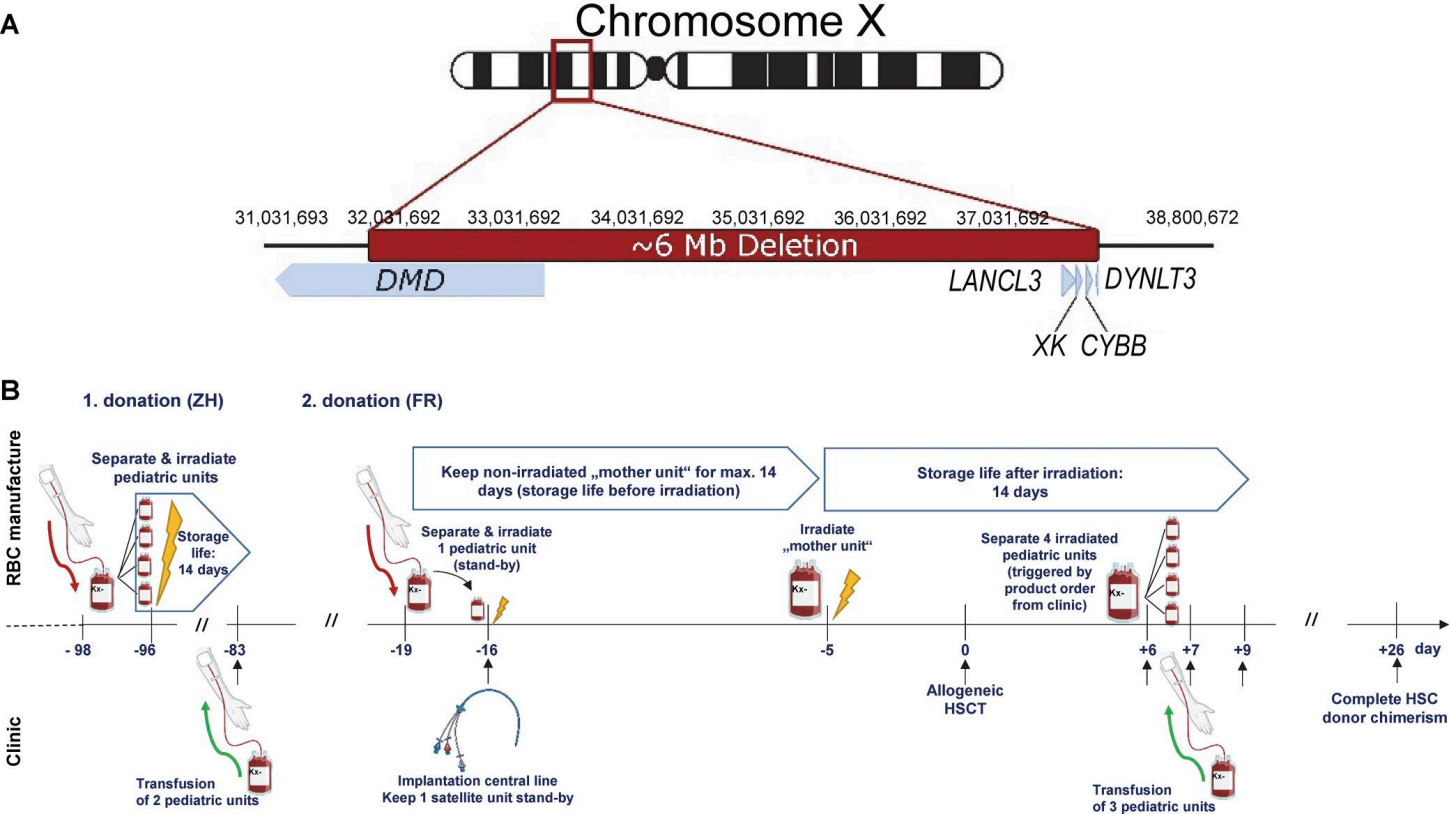
**Large deletion in the X-chromosome and the supply concept with non-cryopreserved Kx− RBCs. (A)** Schematic representation of the large, previously unknown ∼6-Mb deletion identified by stepwise partitioning of the Xp21.1 region (3). Genes involved by this deletion and their orientation of transcription are displayed as blue arrows. **(B)** Managing the supply with Kx− RBCs; ZH: Zurich, Switzerland; FR: Freiburg, Germany. Cartoons were created with BioRender.

Genetic analysis (3) showed a large (5.987.743 bp) deletion (GRCh38/hg38: NC_000023.11:g. 31′884′229_37′871′972del), which was not described before, from intron 47 of *DMD* to intergenic region between *DYNLT3* and *SYTL5* (Fig. 1 A). In total, 12 protein-coding genes were affected (*DMD*, *FAM47A*, *TMEM47*, *FAM47B*, *MAGEB16*, *CFAP47*, *FAM47C*, *PRRG1*, *LANCL3*, *XK*, *CYBB*, and *DYNLT3*).

Currently, HSCT is the only curative treatment for X-linked CGD, with overall survival rates of more than 90% in children. After ethics board consultation and approval, allogeneic HSCT was scheduled. In contrast to previous reports of HSCT, where autologous RBCs could be collected from patients with CGD of 2 and 13 year of age, respectively (2, 4), the transfusion regimen for the herein presented infant needed to rely completely on allogeneic Kx− blood due to the patient’s low blood volume and the need for rapid HSCT.

In the critical phase 3 mo prior to HSCT, the patient had a transfusion-dependent anemia. Therefore, a transfusion regimen with Kx− RBCs from nearly 100 days before HSCT as well as over the entire peritransplant phase needed to be established. No Kx− and RhD− donors were identified by international rare donor programs.

Specifically, the following points needed consideration: (1) the minimal interval between blood donations (55 days), (2) the storage life of the Kx− RBCs (14 days after irradiation), (3) the production of the satellite Kx− RBC units, and (4) the projected clinical interventions and potential need for Kx− RBC transfusions (Fig. 1 B). The Blood Transfusion Service Zurich, Switzerland, had previously identified individuals with MLP without hematologic, neuromuscular, or cerebral involvement and offered support. After confirming donor’s eligibility, consent, and availability, the first blood collection was performed in Zurich, Switzerland, where four irradiated Kx− RBC pediatric (satellite) units (70 ml/U) were produced and shipped to Freiburg, Germany. Of these, two units were transfused (11 ml/kg) 3 mo prior to HSCT. For the second donation prior to HSCT, the donor agreed to travel to Freiburg, Germany, where a regular Kx− RBC “mother unit” was produced, and one satellite bag was immediately separated, irradiated, and kept on standby for central line implantation at day −16. The remaining nonirradiated Kx− RBC unit was stored at +4°C.

The patient received HSCT (bone marrow) from a 10/10 HLA-matched unrelated donor at the age of 7 mo after reduced toxicity conditioning with targeted busulfan (6 × 2.55 mg/kg), fludarabine (5 × 30 mg/m^2^), and alemtuzumab (3 × 0.2 mg/kg) (5). Busulfan area under the curve target was higher than usual (80–90 mg/liter × h), as graft failure is more common in smaller children, and to avoid mixed chimerism. To reduce risk of alloimmunization to RBCs, B cell–depleting therapy with rituximab was given on day −12 and −1 (5). Posttransplant autoimmune hemolytic anemia may occur in this setting and could increase the transfusion need, which should be avoided in this setting with very limited availability of Kx− RBCs. We decided to start the rituximab treatment on day −12 to target recipient B cells, to avoid the first dose on a weekend. This procedure was generally in line with Hönig et al. (4) except that our conditioning did not include total body irradiation (TBI), which we considered too toxic at this age. To specifically target donor lymphocytes to avoid hemolysis of residual recipient red blood cells and immunization in case of mixed chimerism, we intensified the B cell–depleting therapy with a second rituximab dose on day −1. At day −5, the stored Kx− RBC mother unit was irradiated for its availability for the following 14 days peritransplant. The patient received a bone marrow graft at day 0 after complete plasma separation with 6.79 × 10^8^ nucleated cells per kg body weight (BW) and 1.38 × 10^7^ CD34^+^ cells per kg BW.

At day +6, triggered by the first posttransplant RBC order from the clinic, four irradiated pediatric (satellite) units (66 ml/U) were separated from the stored irradiated Kx− RBC unit, of which three (8 ml/kg) were transfused at day +6, +7, and +9, respectively.

To prevent graft-versus-host disease (GvHD), cyclosporine A was given and tapered after day +100 together with mycophenolate mofetil, which was stopped at day +30. No signs of GvHD were present. The patient showed timely engraftment of neutrophils on day +16 and platelets on day +28. Oxidative burst was normal on day +26 and +61, and chimerism in peripheral blood was 100% donor. The patient showed severe veno-occlusive disease. Abdominal ultrasound revealed retrograde flow in the portal vein on day +13 in combination with weight gain, painful hepatomegaly, ascites, and increased platelet consumption, but without hepatic failure. The patient received defibrotide from day +13 to +27 and the symptoms completely normalized, including normal portal venous flow. Reduced kidney function required diuretic medication, and the patient showed mild engraftment syndrome with need for high flow nasal cannula support, responding well to prednisone treatment, which could be tapered quickly and stopped on day +47. The patient was discharged on day +68. Immunological reconstitution was slow with delayed B cell reconstitution, a known complication of peritransplant rituximab use, and required monitoring of residual immunoglobulin levels and supplementation. However, in the benefit-risk-calculation for this patient, rituximab was clearly beneficial. T cell reconstitution was also slow, but not markedly delayed as expected after alemtuzumab treatment. Of note, erythropoiesis recovered quickly, and only three pediatric RBC units had to be transfused without signs of hemolysis after HSCT.

The patient was discharged with complete chimerism at day +68 and was well and remained independent of transfusions and with complete chimerism at last follow-up 2.8 years after HSCT without signs of GvHD. Screening after HSCT was negative for irregular red blood cell antibodies.

## Conclusion

The MLP poses a substantial challenge for blood management, especially in the context of HSCT, where timing of complex procedures, availability of compatible stem cell and blood donors, quality impairment after cryopreservation, logistics, and storage life of RBCs must be considered. Close international collaborations ensured successful HSCT of the young patient, including pre- and post-HSCT transfusions of non-cryopreserved Kx− RBCs. Our approach could serve as a blueprint for assuring a readily available provision of non-cryopreserved Kx− RBCs even over several months in complex clinical settings.
